# Comparison of the effect of spinal anesthesia and general anesthesia on 5-year tumor recurrence rates after transurethral resection of bladder tumors

**DOI:** 10.18632/oncotarget.21034

**Published:** 2017-09-16

**Authors:** Woo-Jong Choi, Seunghee Baek, Eun-Young Joo, Syn-Hae Yoon, Eunkyul Kim, Bumsik Hong, Jai-Hyun Hwang, Young-Kug Kim

**Affiliations:** ^1^ Department of Anesthesiology and Pain Medicine, Asan Medical Center, University of Ulsan College of Medicine, Seoul, Republic of Korea; ^2^ Department of Clinical Epidemiology and Biostatistics, Asan Medical Center, University of Ulsan College of Medicine, Seoul, Republic of Korea; ^3^ Department of Urology, Asan Medical Center, University of Ulsan College of Medicine, Seoul, Republic of Korea

**Keywords:** transurethral bladder tumor resection, tumor recurrence, spinal anesthesia, general anesthesia

## Abstract

Non-muscle invasive bladder tumors are early-stage tumors with high recurrence rates. Transurethral resection of bladder tumor (TURB) is performed under spinal or general anesthesia; however, the effect of the two anesthetic techniques on non-muscle invasive bladder tumor recurrence is unknown. Thus, we compared their effects on tumor recurrence rates five years after TURB. Data from 876 patients who underwent TURB from 2000 to 2007 was reviewed. Patients received spinal or general anesthesia based on their choice or the clinician's preference. Tumor recurrence five years after TURB was assessed using multivariate Cox regression model, propensity score analysis (matching and inverse probability of treatment weighting), and Kaplan–Meier method. The five-year tumor recurrence rate after TURB was 42% and 53% for spinal and general anesthesia groups, respectively (*P* = 0.013). Hazard ratios for tumor recurrence in the spinal anesthesia group compared to that in the general anesthesia group were 0.619 (P <0.001), 0.642 (P = 0.001), and 0.636 (P <0.001) in the Cox regression model, Cox regression model with propensity score matching, and adjusted analysis with inverse probability of treatment weighting, respectively. The five-year tumor recurrence rate was significantly lower in the spinal anesthesia group than in the general anesthesia group in both the unadjusted (P = 0.002) and adjusted Kaplan–Meier curves (P <0.001). Therefore, spinal anesthesia for non-muscle invasive bladder tumor resection was associated with a lower five-year tumor recurrence rate than general anesthesia. This finding provides useful information for an appropriate selection of anesthetic technique for TURB.

## INTRODUCTION

Approximately 70% of bladder tumors are superficial and non-muscle invasive at diagnosis [1]. The primary treatment for non-muscle invasive bladder tumor is transurethral resection by cystoscopy under general or regional anesthesia, which is determined by the clinician's or patient's preference. However, despite additional treatment with bacillus Calmette-Guerin (BCG) immunotherapy [2], the recurrence rate of non-muscle invasive bladder tumor is relatively high [3]. Although the risk factors of tumor recurrence after transurethral resection of bladder tumor (TURB) have been previously described [4], the effect of anesthetic techniques on tumor recurrence after TURB remains unclear.

Tumor recurrence after oncologic surgery can be affected by perioperative factors such as anesthetic techniques, which influence the patient's immune function [5–7]. Regional anesthesia reportedly reduces tumor recurrence because it provides pain relief without the administration of opioids, decreases sympathetic nerve activity during surgery, and reduces perioperative immunosuppression [8, 9]. However, few studies have examined the effect of anesthesia methods in early-stage cancer surgery. The choice of anesthetic techniques is important in early-stage cancer surgery because a complete cure is critical for preventing progression to advanced-stage cancer. Thus, the effect of anesthetic techniques on tumor recurrence should be evaluated in patients undergoing early-stage cancer surgery such as TURB.

Herein, we compared the effect of spinal and general anesthesia on tumor recurrence five years after TURB in early-stage cancer patients with non-muscle invasive bladder tumors by using multivariate Cox regression model, propensity score analysis, and Kaplan–Meier method.

## RESULTS

**Figure 1:**
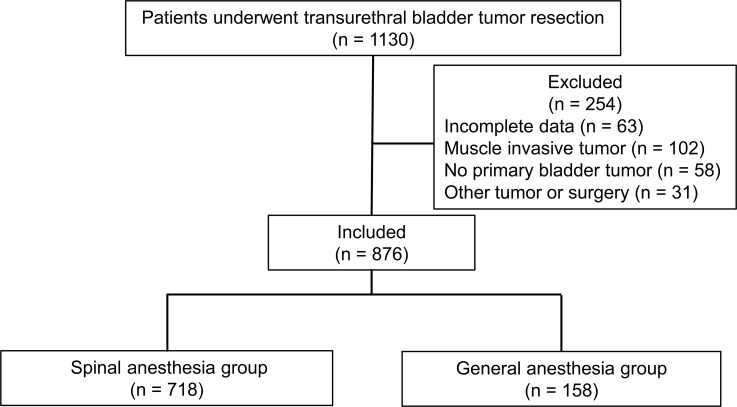
Study flowchart

Medical records of 1130 patients were reviewed; 254 patients were excluded from the present study due to incomplete patient records, muscle invasive bladder tumors, absence of primary bladder tumors, or any other tumors and operations during the study period (Figure 1). Therefore, 876 patients were included in the present study.

The median follow-up time was 35 months (interquartile range: 11–57 months). Table 1 shows the demographic and perioperative characteristics of all patients. There were significant differences in age between the two groups before propensity score matching. To reduce the confounding effects due to the differences in baseline characteristics associated with anesthetic technique allocation, all variables were balanced between the two groups after 1:4 propensity score matching or incorporation with inverse probability of treatment weighting (IPTW). Finally, 690 patients (spinal: general = 534: 156) were obtained after 1:4 matching on the propensity score. Balance between groups was improved after matching, and standardized differences were less than 0.1 for all variables (Table 2).

**Table 1:** Patient characteristics before propensity score matching and weighting

	Spinal anesthesia (n = 718)	General anesthesia (n = 158)	Standardized difference	*P*
Age (y)	63 ± 12	61 ±13	0.129	0.043
Gender				0.186
Male	598 (83%)	124 (78%)	0.122	
Female	120 (17%)	34 (22%)	−0.122	
ASA physical status				0.582
I	232 (32%)	49 (31%)	0.028	
II	451 (63%)	104 (66%)	−0.063	
III	38 (5%)	5 (3%)	0.087	
Tumor size				0.109
<1 cm	178 (25%)	52 (33%)	−0.180	
1-3 cm	398 (55%)	79 (50%)	0.109	
>3 cm	142 (20%)	27 (17%)	0.069	
Tumor number				0.164
Single	342 (48%)	65 (41%)	0.131	
Multiple	376 (52%)	93 (59%)	−0.131	
Tumor grade				0.330
1	113 (16%)	29 (18%)	0.043	
2	360 (50%)	79 (50%)	−0.043	
3	245 (34%)	50 (32%)	−0.130	
Tumor stage				0.252
Ta	447 (62%)	90 (57%)	0.043	
T1	271 (38%)	68 (43%)	−0.043	
Carcinoma *in situ*, n (%)	54 (8%)	10 (6%)	0.089	0.725
BCG therapy, n (%)	278 (39%)	58 (37%)	0.041	0.659
Chemotherapy, n (%)	154 (21%)	27 (17%)	0.111	0.517

Data are presented as mean ± SD or number (percentage). ASA = American Society of Anesthesiologists; BCG = bacillus Calmette-Guerin.

**Table 2:** Patient characteristics after propensity score matching (1:4 matching) and weighting

	Spinal anesthesia (n = 534)	General anesthesia (n = 156)	Standardized differences	Adjusted *P*	*P* with IPTW
Age (y)	62 ± 12	61 ± 13	0.087	0.173	0.322
Gender				0.544	0.881
Male	436 (82%)	124 (79%)	0.055		
Female	98 (18%)	32 (21%)	−0.055		
ASA physical status				0.914	0.970
I	165 (31%)	49 (31%)	−0.011		
II	348 (65%)	102 (65%)	−0.005		
III	21 (4%)	5 (3%)	0.039		
Tumor size				0.455	0.981
<1 cm	147 (28%)	51 (33%)	−0.013		
1-3 cm	287 (54%)	78 (50%)	0.075		
>3 cm	100 (19%)	27 (17%)	0.037		
Tumor number				0.787	0.928
Single	229 (43%)	65 (42%)	0.025		
Multiple	305 (57%)	91 (58%)	−0.025		
Tumor grade				0.892	0.883
1	91 (17%)	29 (19%)	−0.040		
2	265 (50%)	77 (49%)	0.005		
3	178 (33%)	50 (32%)	0.027		
Tumor stage				0.467	0.897
Ta	322 (60%)	89 (57%)	0.066		
T1	212 (40%)	67 (43%)	−0.066		
Carcinoma *in situ*, n (%)	39 (7%)	10 (6%)	0.035	0.702	0.842
BCG therapy, n (%)	200 (37%)	56 (36%)	0.032	0.723	0.717
Chemotherapy, n (%)	105 (20%)	27 (17%)	0.061	0.511	0.932

Data are presented as mean ± SD or number (percentage). ASA = American Society of Anesthesiologists; BCG = bacillus Calmette-Guerin; IPTW = inverse probability of treatment weighting.

In multivariable Cox regression model, spinal anesthesia, age, tumor size >3 cm, multiple tumor, BCG therapy, and chemotherapy were significantly associated with tumor recurrence in all patients. The hazard ratio for tumor recurrence in the spinal anesthesia group compared with that in the general anesthesia group was 0.619 (*P* < 0.001) in the Cox regression model and 0.642 (*P* = 0.001) in the Cox regression model with propensity score matching (Table 3). Additionally, in the adjusted analysis with IPTW, the risk of tumor recurrence in the spinal anesthesia group was significantly lower than that in the general anesthesia group, with a hazard ratio of 0.636 (*P* < 0.001) (Table 3).

**Table 3:** The impact of spinal anesthesia on tumor recurrence

	Multivariate Cox regression model^*^		PS matching^*^		IPTW^*^	
	HR (95% CI)	*P*	HR (95% CI)	*P*	HR (95% CI)	P
Tumor recurrence	0.619 (0.484–0.793)	<0.001	0.642 (0.498–0.826)	0.001	0.636 (0.502–0.806)	<0.001

PS = propensity score; IPTW = inversed probability of treatment weighting; HR = hazard ratio; CI = confidence interval. ^*^ Adjusted by all variables in Table 1.

**Figure 2:**
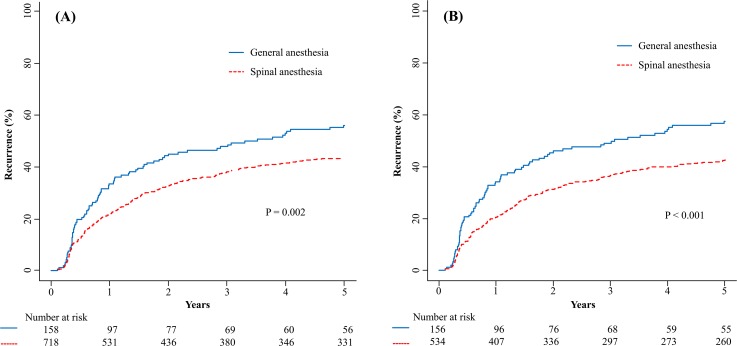
Kaplan–Meier curves of the five-year tumor recurrences after transurethral resection of bladder tumors in patients who received either spinal or general anesthesia before **(A)** and after **(B)** adjustment by propensity score matching.

The five-year tumor recurrence rate after TURB was 42% and 53% for spinal and general anesthesia group, respectively (*P* = 0.013). The mean time to tumor recurrence was 41.1 ± 0.85 and 35.5 ± 1.85 months for the spinal and general anesthesia groups, respectively (*P* < 0.001). Figure 2 shows Kaplan–Meier curves for the time to bladder tumors recurrence in the spinal and general anesthesia groups. The five-year tumor recurrence rate in the spinal anesthesia group was significantly lower than that in the general anesthesia group in both the unadjusted (*P* = 0.002) (Figure 2A) and adjusted Kaplan–Meier curves in the matched samples (*P* < 0.001) (Figure 2B).

## DISCUSSION

In non-muscle invasive bladder tumors, the five-year tumor recurrence rates after TURBs were lower in patients who underwent surgery with spinal anesthesia than in patients who underwent surgery with general anesthesia. After adjustment of confounding factors using propensity score analysis, we found that patients who received spinal anesthesia had a significantly lower risk of tumor recurrence than those who received general anesthesia. Therefore, spinal anesthesia may be an important variable for the five-year tumor recurrence risk after non-muscle invasive bladder tumor surgery.

When evaluating the influence of anesthesia on oncologic outcomes, factors such as use of systemic opioids, regional anesthesia, and exposure to inhaled anesthetics need to be considered. Systemic opioids decrease both humoral and cellular immunity [10–12], and previous studies reported that the use of regional anesthesia has been related with improved oncologic outcomes due to the decrease of systemic opioid usage [13–15]. Regional anesthesia also attenuates the release of stress hormones and sympathetic nerve activation [16–18]. Furthermore, several studies reported that epidural anesthesia combined with general anesthesia and epidural analgesia might improve oncologic outcomes in gastric cancer surgery [19] and resection of colorectal liver metastases [20]. In the present study, no systemic opioids were used in either the general or regional anesthesia groups because TURB is not a very painful procedure. Therefore, we were able to isolate and evaluate the effect of regional versus general anesthesia in non-muscle invasive bladder tumor surgery.

Exposure to inhaled anesthetics has been known to have a prolonged and profound inhibitory influence on natural killer cell activity [21]. Hole et al. [22] evaluated monocyte function in patients undergoing total hip arthroplasty performed under either general or epidural anesthesia. Although the phagocytic function of the monocytes was reported to be improved in the epidural group, a decrease in monocyte phagocytosis was observed in the general anesthesia group. In addition, Wada et al. [23] demonstrated that laparotomy under sevoflurane anesthesia diminished the tumorcidal activity in liver mononuclear cells (T-helper cells) in a rat model. Thus, the induction of general anesthesia by inhaled anesthetics may be associated with immunosuppression and host defense impairment, which can result in the acceleration of tumor growth [12, 24]. Therefore, considering all the components of the present study, it appears that the lack of exposure to inhaled anesthetics can be a key component in the positive relationship between spinal anesthesia and improved oncologic outcomes after TURB.

Non-muscle invasive bladder tumors are early-stage tumors, but their recurrence rate is high after initial transurethral resection. This may stem from oversight or incomplete removal of tumor cells during resection. Thus, immunotherapy with BCG or chemotherapy with mitomycin C has been employed to reduce the risk of tumor recurrence and progression in patients with high-risk non-muscle invasive bladder tumors [2]. Chemotherapy with mitomycin C is administered to kill residual tumor cells via intravesical instillation. Immunotherapy with BCG is specifically designed to boost the host's natural defences to fight the tumor. BCG attaches to the inner lining of the bladder, thereby attracting immune cells to the area to fight the tumor cells [25]. Therefore, both immunotherapy with BCG and chemotherapy with mitomycin C may be important confounding variables in the present study. However, we concluded that neither could have significantly affected our results because the distribution of these therapies was statistically similar in patients who received spinal or general anesthesia.

This retrospective study is limited by the non-randomized assignment to the anesthetic groups and the difficulty of controlling bias. We cannot rule out the possibility that the between-group differences may be due, at least in part, to the differences in the confounding factors, even if the propensity score analysis was applied.

In conclusion, spinal anesthesia for non-muscle invasive bladder tumor resection was associated with a lower tumor recurrence rate than general anesthesia, after adjusting for confounding variables. These data provides valuable information on the association between the anesthetic technique and surgical outcome in patients with early-stage bladder tumors, especially those who undergo TURB.

## MATERIALS AND METHODS

### Data source and selection criteria

This study was approved by the Institutional Review Board of Asan Medical Center (number: 2013-0067). The patients were selected from the clinical registry maintained by the Department of Anesthesiology. The data from all patients who underwent TURB between January 2000 and December 2007 were reviewed. In this study, we included all patients diagnosed with non-muscle invasive bladder tumors by TURB and biopsy.

After TURB, intravesical therapy with BCG was administered in cases of high-risk non-muscle invasive bladder tumors [1]. In a select group of patients, chemotherapy with mitomycin C was carried out in a perioperative manner via intravesical delivery immediately after TURB.

Patients diagnosed with muscle invasive bladder tumors by TURB and biopsy were excluded from this study because they underwent radical bladder resection and/or bladder reconstruction under general anesthesia. We also excluded patients with incomplete medical records, no primary bladder tumor, or any other tumors or surgeries during the follow-up period.

### Data collection

From the patients’ medical records, we extracted data on demographic characteristics and tumor-related factors: age, gender, anesthesia technique, American Society of Anesthesiologists physical status, tumor size, tumor number, tumor grade, tumor stage, carcinoma *in situ*, intravesical BCG treatment, and chemotherapy. Follow-up evaluations were performed at three-month intervals for the first two years, followed by evaluations every six months for the next three years, and then annually afterwards.

### Definition of tumor recurrence

Tumor recurrence after TURB was determined by clinical symptoms at follow-up or spontaneous consultation, and confirmed by the detection of new bladder tumors in cystoscopy or urinalysis. The time to tumor recurrence was defined as the time from the date of the operation to tumor recurrence.

### Anesthetic techniques

All patients included in this study underwent TURB under either general or spinal anesthesia. Patients received general or spinal anesthesia based on the patient's choice and the clinician's preference. General anesthesia was induced with thiopental (4–5 mg/kg) and rocuronium (0.5–0.6 mg/kg) or atracurium (0.5 mg/kg). Laryngeal mask airway was inserted after muscle paralysis. General anesthesia was maintained with nitrous oxide (50%) and sevoflurane (1–3 vol%). At our institution, no analgesics or opioids were administered intraoperatively as this was not a severely painful procedure. For spinal anesthesia, 10–12 mg of 0.5% heavy bupivacaine was administrated intrathecally. Midazolam (2–5 mg) was administrated intravenously at the patient's request for sedation. Intravenous paracetamol 1000 mg per 6 h was administered to control postoperative pain in both general and spinal anesthesia groups.

### Statistical methods

The basic characteristics and confounding variables of spinal and general anesthesia groups were compared using the chi-square test or Fisher's exact test for categorical variables and the Student's t test for age. Time to recurrence was calculated from the date of operation to recurrence. For patients who had not experienced the event of interest, the respective event time was censored at the time of the last urologic follow-up or death not due to the event of interest. Tumor recurrence after TURB was evaluated using the Kaplan–Meier method.

Because patients were not randomly allocated to the two anesthetic groups, propensity score was used to decrease the potential bias and to render the two groups more comparable. The propensity score was defined as the conditional probability of receiving spinal anesthesia given a set of measured variables. To estimate the propensity score, logistic regression model was applied, and anesthetic techniques (spinal anesthesia vs. general anesthesia) were regressed on the baseline variables (Table 1).

Propensity score analysis was implemented in two ways to control for confounding variables: propensity score matching and propensity score weighting. Propensity score matching was performed using a one-to-four nearest neighbor matching with replacement with a caliper size of 0.1 standard deviations (R library ‘MatchIT’). Balances in the distribution of baseline covariates were evaluated by calculating the absolute standardized differences of the covariates between the two groups before and after matching. As the propensity score-matched sample does not consist of independent observations, we applied a marginal survival model with robust standard errors. Propensity score weighting was performed by the IPTW derived from the propensity score. If a subject had a higher probability of being in a group, it was regarded as being overrepresented and was allocated with a lower weight. On the contrary, if the subject had a smaller probability, it was regarded as being underrepresented and was allocated with a higher weight. To decrease the effect of outlying weights, weights were stabilized via multiplication by the mean propensity score of the known exposure group [26]. The variables between the two groups after 1:4 propensity score matching were compared using the chi-square test or Fisher's exact test for categorical variables and the Student's t test for age. After performing propensity score weighting, we compared the variables between the two groups using the weighted t test for age and the weighted chi-square test for categorical variables. We then fitted weighted Cox proportional hazards model using a group indicator variable of anesthetic techniques. Statistical analysis was performed using R 3.3.1 (http://www.r-project.org) with packages of ‘MatchIt’ and ‘weights’.
